# Block-Adaptive Rényi Entropy-Based Denoising for Non-Stationary Signals

**DOI:** 10.3390/s22218251

**Published:** 2022-10-28

**Authors:** Nicoletta Saulig, Jonatan Lerga, Siniša Miličić, Željka Tomasović

**Affiliations:** 1 Faculty of Engineering, Juraj Dobrila University of Pula, 52100 Pula, Croatia; 2 Faulty of Engineering, University of Rijeka, 51000 Rijeka, Croatia; 3 Center for Artificial Intelligence and Cybersecurity, University of Rijeka, 51000 Rijeka, Croatia; 4 Faulty of Informatics, Juraj Dobrila University of Pula, 52100 Pula, Croatia

**Keywords:** non-stationary signal, time-frequency distribution, denoising, Rényi entropy

## Abstract

This paper approaches the problem of signal denoising in time-variable noise conditions. Non-stationary noise results in variable degradation of the signal’s useful information content over time. In order to maximize the correct recovery of the useful part of the signal, this paper proposes a denoising method that uses a criterion based on amplitude segmentation and local Rényi entropy estimation which are limited over short time blocks of the signal spectrogram. Local estimation of the signal features reduces the denoising problem to the stationary noise case. Results, presented for synthetic and real data, show consistently better performance gained by the proposed adaptive method compared to denoising driven by global criteria.

## 1. Introduction

Non-stationary signals, produced by numerous phenomena that are the focus of various disciplines of engineering, demand particular techniques of representation and analysis due to their frequency content, which varies over time [1].

Two-dimensional energy time-frequency distributions (TFDs) have been found to be particularly useful tools when dealing with challenging features, such as multiple components presenting variable instantaneous frequencies (IFs), or unwanted presence of noise.

In various engineering applications, what is often considered the useful information content of a signal consists of signal components represented in the time-frequency (TF) plane by continuous energy regions, peaks of which correspond to the IFs of the noise-free signal.

However, degradation of the signal quality, which may occur due to noise present during the signal acquisition or transmission, makes the extraction of the signal components from the background noise a challenging task.

A variety of scientific and engineering applications are focused on the extraction of the signal’s components from the noisy mixture [2,3,4,5,6,7,8].

Approaches that make use of knowledge of specific signal patterns have been applied to particular signal categories, such as EEG, seismic signals, and speech [9,10,11,12,13,14,15].

On the other hand, methods that are not limited to particular types of signals have been proposed, but these often put constraints on the signal structure (a constant or known number of components is required in the entire measurement time, or signals components are not allowed to intersect) [16,17,18,19].

Selection of the TFD regions that support the signal components have also been proposed by thresholding methods (usually as a percentage of the maximal TFD value) [2,4,7]. However, if the choice of the threshold is not driven by trial-and-error procedures, it can produce significant errors in the extracted information.

New insights into the TFD structure have been provided by local TF entropy analysis. In [20], a TFD amplitude segmentation is proposed to partition the TFD into classes, whose local Rényi entropies (LRE) are evaluated and compared in order to select only those classes that contain the signal components. Compared to methods that make use of 2-D entropy maps for signal denoising [21], the LRE approach, which is a 1-D estimation, drastically reduces the computational cost.

In this paper, we propose a 1-D entropy-based method that, unlike the one in [20], is adapted to perform in conditions of variable noise intensity. This is possible due to multiple partitioning of the signal TFD; the TFD is initially divided into time building blocks, and each block is then subject to amplitude segmentation. The method is not limited to certain types of signals and does not require any previous knowledge of the signal.

## 2. Materials and Methods

### 2.1. Background Theory

A non-stationary multi-component signal can be written in the form [1]

$$x\left(t\right)=\sum_{l=1}^{L}x_{l}\left(t\right)=\sum_{l=1}^{L}A_{l}\left(t\right)e^{j\Phi_{l}\left(t\right)},$$

with *L* being the total number of signal components, $A_{l}\left(t\right)$ the amplitude of each of the individual components $x_{l}\left(t\right)$, and $\Phi_{l}\left(t\right)$ the instantaneous phase. The IF corresponds to the derivative of the instantaneous phase as $f_{l}\left(t\right)=\Phi_{l}^{\prime }\left(t\right)/2\pi$.

Such signals, however, may be distorted at the time of their collection from noisy environments, as well as during the transmission process.

An additive signal model that takes into account the presence of independent additive white Gaussian noise (AWGN), ν(t)∼N(0,1), reads

(1) $$y\left(t\right)=x\left(t\right)+\sigma_{\nu }\nu \left(t\right).$$

On the other hand, more complex noise conditions may occur as in the case of non-stationary noise, which may be the result of noise intensity modulation. In this case, the noise is of the form w(t)ν(t), with ν(t) being unit normal distribution variance and w(t) non-constant positive. For any $t_{1},t_{2}$, the random variable

(2) $$\begin{array}{c} \frac{1}{\sqrt{w^{2}\left(t_{1}\right)+w^{2}\left(t_{2}\right)}}\left(w\left(t_{1}\right)\nu \left(t_{1}\right)+w\left(t_{2}\right)\nu \left(t_{2}\right)\right) \end{array}$$

is the unit normal distribution, hence the process is Gaussian, but, since the amplitude of the process w(t) varies, the process is not stationary in either a strict or weak sense. Thus, even in an ostensibly Gaussian case, non-trivial effects may occur.

The simultaneous presence of multiple spectral components and noise privileges representations of such signals in the joint time-frequency domain.

The general class of quadratic time-frequency distributions (TFDs) is defined in terms of the noisy signal instantaneous autocorrelation function, and the time-lag kernel G(t,τ), as [1]

(3) $$\begin{array}{c} \rho_{y}\left(t,f\right)=\int_{-\infty }^{\infty }\int_{-\infty }^{\infty }G\left(t-u,\tau \right)\;y\left(u+\frac{\tau }{2}\right)\;y^{★}\left(u-\frac{\tau }{2}\right)e^{-j2\pi f\tau }\;du\;d\tau . \end{array}$$

A discrete model of the noisy signal reads

(4) $$\begin{array}{c} y(n)=x(n)+\nu (n), \end{array}$$

where

(5) $$\begin{array}{c} x\left(n\right)=\sum_{l=1}^{L}x_{l}\left(n\right)=\sum_{l=1}^{L}A_{l}\left(n\right)e^{j\Phi_{l}\left(n\right)}. \end{array}$$

The discrete quadratic class TFD is computed as the discrete Fourier transform (DFT) of the convolution of the time-lag kernel filter G(n,i) and the instantaneous autocorrelation function as [1]

(6) $$\begin{array}{c} \rho \left(n,m\right)=\underset{i\to m}{\mathrm{DFT}}\left\{G\left(n,i\right)\underset{n}{*}\left(y\left(n+i\right)y^{★}\left(n-i\right)\right)\right\}, \end{array}$$

for *i* in an interval of integers.

In the attempt to select the TFD regions that are considered useful for further analysis, i.e., signal component, data amplitude can be considered as a discriminant. In fact, signal components, when represented in the TF domain, tend to be prominent energy ridges emerging from the noisy background. However, establishing a threshold value to discriminate useful data from noise appears far from practicable.

In this sense, machine learning methods can be considered useful tools for initial data segmentation.

Amplitude discrimination of data in the TFD can be achieved by application of the *K*-means algorithm [22]. If the TFD, ρ(n,m), is considered as an N×M-dimensional set of observations, the K-means algorithm partitions these N×M observations into *K* sets

(7) $$\begin{array}{c} C=\{C_{k}|k\in N,\;1\leq k\leq K\}, \end{array}$$

by minimizing the within-cluster sum of squares as:

(8) $$\begin{array}{c} \underset{C}{\mathrm{argmin}}\;\sum_{k=1}^{K}\sum_{\left(n,m\right)\in C_{k}}^{}{∥\rho \left(n,m\right)-P_{k}∥}^{2}, \end{array}$$

where $P_{k}$ is the mean of each set $C_{k}$.

Thus *K* classes $\rho_{k}\left(n,m\right),k\in N,\;1\leq k\leq K$, are obtained from the TFD as collection of coefficients ρ(n,m) satisfying

(9) $$\begin{array}{c} \rho_{k}\left(n,m\right)=1_{C_{k}}\left(n,m\right)\rho \left(n,m\right), \end{array}$$

with the set indicator function defined by

(10) $$\begin{array}{c} 1_{S}\left(X\right)=\left\{\begin{array}{cc} 1, & X\in S\\ 0, & \mathrm{otherwise}. \end{array}\right. \end{array}$$

Yet, the segmentation procedure itself can not be considered a predictor of the origins of data contained in a particular class. Namely, classes containing mainly noise-originated data should be discarded from further analysis, while classes containing data signal components should be preserved.

The white noise assumption predicts noise power spectral density evenly spread over the signal bandwidth. In fact, a TFD of dimension *N* computed over *M* frequency bins, with *L* components supported over 1-D trajectories, with L≪N, imposes a sparse distribution on the TF plane [23].

In light of the above, classes containing mainly noise coefficients are expected to present large frequency supports (intended as the subset of the frequency domain containing non-zero elements), in comparison to classes containing signal components.

As a result, the segmentation procedure, ruled by Exp. (8), assigns densely distributed classes to components, due to their fast amplitude changes. Accordingly, data in the resulting classes will present fractional frequency supports in comparison to classes populated by noise-originated data.

### 2.2. A LRE-Based Criterion for Useful Class Selection

In [20], the properties of the LRE, well-known measures of signal supports in the TF plane, have been exploited in order to identify structural differences between the noise classes and signal component classes.

The LRE is obtained by estimating the Rényi entropy over a short time slice of duration Δn of one class of a positive TFD, as:

(11) $$H_{k}\left(p\right)=\frac{1}{1-\alpha }\log_{2}\sum_{n,m}{\bar{\rho }}_{k}{\left(n,m\right)}^{\alpha }$$

with *n* constrained to the interval [p−Δn/2,p+Δn/2], *m* going over all frequency bins, and $\bar{\rho }\left(m,n\right)$ normalized to 1 across the computation domain.

The LRE measure, highly indicative of TFD supports, is then used as input to a selection procedure that provides classes containing signal components. This procedure produces highly reliable discrimination of classes containing noise from classes containing signal components.

**Figure 1:**
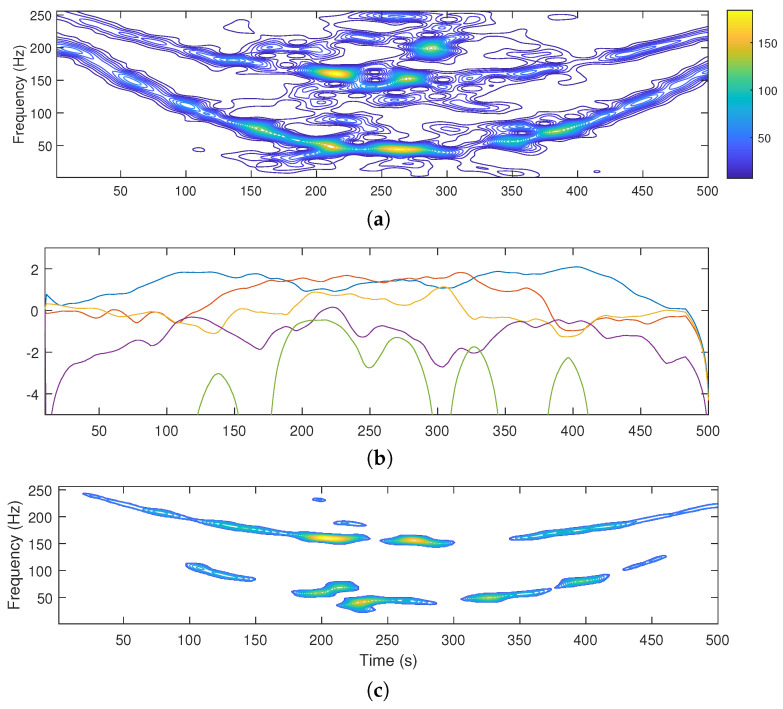
TFD of a noisy signal (**a**), LRE estimates for K=5 classes (1. class blue, 2. class red, 3. class orange, 4. class purple, and 5. class green) (**b**), and useful information extracted by the LRE criterion (**c**).

The TFD of a two-component noisy signal and LRE estimates $H_{k}\left(p\right)$ for *K*-means segmentation into five classes are reported in Figure 1a,b.

The selection procedure reported in the steps below is based on a relative distance criterion of the LRE functions $H_{k}\left(p\right)$:

1. The first class $\rho_{1}\left(n,m\right)$, consisting of the smallest coefficients obtained from expression Exp. (8), associated to the LRE function $H_{1}\left(p\right)$, is discarded as noise.
2. Starting from the second class, $\rho_{2}\left(n,m\right)$, all consecutive classes for which for at least one instant *p* $H_{k}\left(p\right)\geq H_{1}\left(p\right)$ holds are classified as noise, and thus discarded.
3. Considering that in the previous steps a total of *r* classes have been discarded, for the remaining classes, $\rho_{k}\left(p\right),r<k\leq K$ we introduce a threshold *i* as follows. We first introduce a closeness value $d_{k}$ on the remaining classes by
  (12) $$d_{k}=\min\{|H_{k}\left(p\right)-H_{k+1}\left(p\right)\left|,\;\mathrm{across}\;\mathrm{all}\;p\right\}.$$
  We now define *i* as the least index of $d_{i}$ such that $d_{k}<d_{i}$ for k<i and $d_{k}\geq d_{i}$ for k>i.
4. The remaining classes, with indexes k=i,...,K, are added up to obtain the useful information content of the signal, i.e., the signal components.

The criterion for class selection requires, according to Exp. (8), that the first class $\rho_{1}\left(n,m\right)$ is populated by coefficients with the smallest amplitudes. By this assumption, the first class is discarded. The algorithm then relies on a class’s structural affinity criterion: if a class, $\rho_{k}\left(n,m\right)$, presents larger frequency supports than $\rho_{1}\left(n,m\right)$, it will be also considered populated by noise coefficients. In other words, all the classes that for at least one instant *p* satisfy $H_{k}\left(p\right)\geq H_{1}\left(p\right)$ are also discarded.

The remaining classes, starting from the two consecutive classes whose LRE presents the smallest difference $H_{k}\left(p\right)-H_{k+1}\left(p\right)$, are classified as useful classes, i.e., containing coefficients generated from signal components.

The useful classes summed up, representing the extracted signal components, are shown in Figure 1c.

However, the method has been initially formulated for noisy signals in the form of Equation (4), which implies constant noise intensity over the measurement time. This fact then puts into question the suitability of the method for a more generalized signal of the form

(13) $$\begin{array}{c} y(n)=x(n)+w(n)\;\nu (n), \end{array}$$

with non-constant noise intensity w(n)≠const.

In fact, in the case of noise with variable intensity, the region of the signal presenting the largest local signal-to-noise ratio (SNR) will be the one dictating the number of classes to be discarded. This will cause an unjustified loss of useful information in the regions of the TF plane where the components’ structure is better preserved.

The described scenario can be observed in Figure 1: the two-component non-stationary signal is embedded in AWGN modulated by a Gaussian window of the form $w\left(n\right)=e^{-2\beta^{2}\frac{{\left(n-N/2\right)}^{2}}{{\left(N-1\right)}^{2}}}$, β=2.5. As can be observed, the intense noise level in the central part of the signal forces classes $C_{1},C_{2}$, and $C_{3}$ to be discarded, since in the central part of the signal noise content is significant. On the other hand, in the initial and final part of the measurement interval, the presence of noise is negligible, which is ignored by the selection criterion. This results in severe loss of signal components in the regions of the TF plane where the level of noise is low.

### 2.3. A Short-Term LRE Approach for Variable Noise Intensity Conditions

In order to address the problem of component extraction in the case of noise with variable intensity over time, we propose a local approach, which accounts for variable degradation of useful content over the temporal axis. To achieve a local insight into the TFD structure, we split the TFD into temporal segments,

(14) $$\begin{array}{c} \rho_{t}\left(n,m\right)=\rho \left(n,m\right)1_{[(t-1)\Delta t,t\Delta t\rangle }\left(n\right), \end{array}$$

representing the TFD as the sum of $\frac{N}{\Delta t}\in N$ blocks of duration Δt.

The amplitude segmentation based on the *K*-means over N/Δt blocks will result in *K* classes for each block:

(15) $$\begin{array}{c} C_{t}=\left\{C_{k,t}|k\in N,\;1\leq k\leq K\right\}. \end{array}$$

Again, the within-cluster sum of squares minimization is applied to produce these *K* classes for each building block, as

(16) $$\begin{array}{c} \underset{C_{t}}{\mathrm{argmin}}\;\sum_{k=1}^{K}\sum_{\left(n,m\right)\in C_{k,t}}^{}{∥\rho_{t}\left(n,m\right)-P_{k,t}∥}^{2}, \end{array}$$

where $P_{k,t}$ is the mean of each set $C_{k,t}$.

Thus we obtain *K* classes $\rho_{k,t}\left(n,m\right),k\in N,\;1\leq k\leq K$, derived form one TFD block $\rho_{t}\left(n,m\right)$, as follows

(17) $$\begin{array}{c} \rho_{k,t}\left(n,m\right)=\rho_{t}\left(n,m\right)1_{C_{k}}\left(n,m\right). \end{array}$$

For a non-negative TDF, the LRE is now well-defined and dependent both on *t*, representing the block index, and *p*, the temporal index *inside* one block:

(18) $$\begin{array}{c} H_{k,t}\left(p\right)=\frac{1}{1-\alpha }\log_{2}\sum_{n,m}{\bar{\rho }}_{k,t}^{\alpha }\left(n,m\right), \end{array}$$

with *n* constrained to the interval [p−Δn/2,p+Δn/2], *m* going over all frequency bins, and where $\bar{\rho }\left(m,n\right)$ is normalized across the summation domain.

Since the local Rényi entropy of Equation (18) is frequency-shift invariant, by shifting different energy regions inside one time slice Δn we can obtain a single energy region with compact frequency support Δm, centered around an arbitrary frequency $\mu_{t}\left(p\right)$.

Taking a non-zero region inside a short time interval Δn and frequency *m*, with frequency support Δm, we approximate all non-zero elements in one class $\rho_{k,t}\left(n,m\right)$ by $A_{k}$. This approximation is justified since the *K*-means clustering is done across amplitudes.

The adopted approximation makes the amplitude $A_{k}$ homogenous of order α in the expression for the Rényi entropy, so it cancels out.

The local Rény entropy of $\rho_{k,t}\left(n,m\right)$ now becomes

(19) $$\begin{array}{c} H_{k,t}\left(p\right)\approx \log_{2}\Delta n\Delta \mu_{t}\left(p\right). \end{array}$$

Thus, regardless of the choice of α, the LRE describes a normalized weight of the TFD domain belonging to a certain amplitude class and point in time dependent on *p* and *t*.

The criterion for selecting classes containing useful information is now applied to each time block *t* ranging from 1 to N/Δt. Thus, the algorithm can be summarized as follows:

1. Initially *t* is set to one.
2. The first class $\rho_{1,t}\left(n,f\right)$, consisting of the smallest coefficients obtained from Exp. (15) and associated with the LRE function $H_{1,t}\left(p\right)$, is discarded as noise.
3. Starting from the second class, $\rho_{2,t}\left(n,f\right)$, all consecutive classes for which for at least one instant *p* holds $H_{k,t}\left(p\right)\geq H_{1,t}\left(p\right)$ are discarded as noise.
4. Assuming that in steps 2 and 3 a total of $r_{t}$ classes has been discarded, for the remaining classes $\rho_{k,t}\left(p\right)$, $r_{t}<k\leq K$ we introduce a threshold $i_{t}$ as follows. We first introduce a closeness value $d_{k,t}$ on the remaining classes by
  (20) $$\begin{array}{c} d_{k,t}=\min\{|H_{k,t}\left(p\right)-H_{k+1,t}\left(p\right)\left|,\;\mathrm{across}\;\mathrm{all}\;p\right\}. \end{array}$$
  We now define $i_{t}$ as the least index of $d_{i_{t},t}$ such that $d_{k,t}<d_{i,t}$ for $k<i_{t}$ and $d_{k,t}\geq d_{i,t}$ for $k>i_{t}$.
5. The classes with indices $k=i_{t},\ldots ,K$ are summed together to obtain the TFD building block with the useful information content of the signal at block *t*,
  (21) $$\begin{array}{c} {\mathrm{UI}}_{t}\left(n,m\right)=\sum_{k=i_{t}}^{K}\rho_{k,t}\left(n,m\right). \end{array}$$
6. *t* is incremented by one and the procedure is repeated from step 2, until t=N/Δt.
7. The total useful information of the signal is obtained by summing the extracted information over all the building blocks as
  (22) $$\begin{array}{c} \mathrm{UI}=\sum_{t=1}^{N/\Delta t}{\mathrm{UI}}_{t}. \end{array}$$

**Figure 2:**
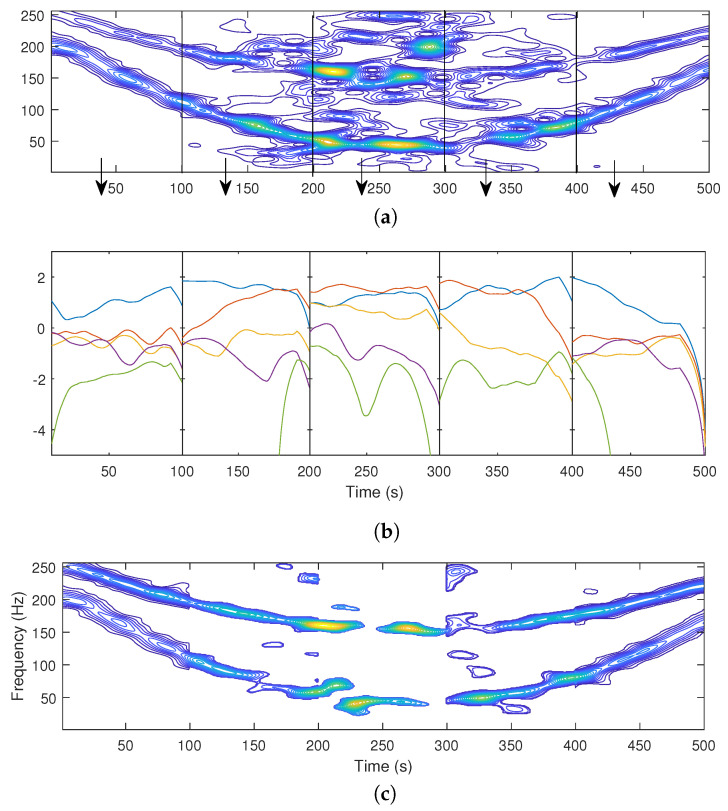
Noisy signal TFD (**a**), LRE estimates over different building blocks for K=5 classes (1. class blue, 2. class red, 3. class orange, 4. class purple, and 5. class green) (**b**), and useful information extracted by the proposed block-adaptive LRE criterion (**c**).

The performance of the proposed method, applied over Δt=N/5 TFD blocks with N=500s, is reported in Figure 2. The noisy TFD is partitioned in five blocks of duration Δt=100s (Figure 2a). The LRE is estimated for five classes of each of the TFD blocks (Figure 2b), and the output of the algorithm according to the selection criterion applied to each of the TFD building blocks is shown in Figure 2c.

Compared to the extracted components when the LRE is estimated over the entire time axis (Figure 1c), the presented approach, which estimates the LRE over individual time blocks, clearly preserves useful information (as visible from the first and last time blocks) by adapting the number of removed coefficients over the measurement time.

## 3. Results

### 3.1. Real Data

As illustrative examples of real-life signals, acoustic waves propagated in air, namely a flute sound signal and a bird song signal, are considered (the omnidirectional microphone used for data collection has a sample/bit rate of 48 kHz/16-bit, frequency response of 20 Hz–20 kHz, and sensitivity −36 dB (1 V/Pa at 1 kHz)).

The flute signal has been collected by a bit depth of 16 bits per sample, with a sampling rate of 8 kHz degraded by transient television static.

**Figure 3:**
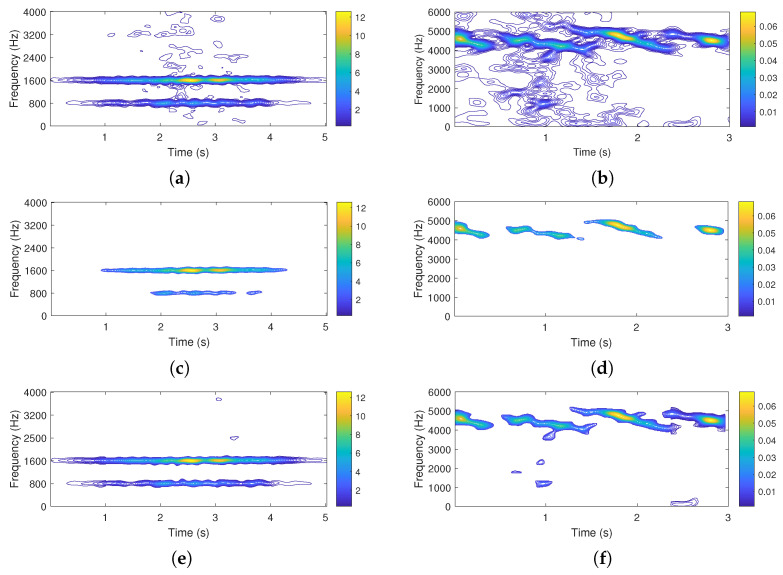
Noisy TFD of a flute sound signal (**a**), noisy TFD of a bird song signal (**a**), extracted signal components of the flute sound by the non-adaptive LRE algorithm (**c**), extracted signal components of the bird song signal by the non-adaptive LRE algorithm (**d**), extracted components of the flute sound signal by the proposed block-adaptive LRE algorithm (**e**), and extracted components of the bird song signal by the proposed block-adaptive LRE algorithm (**f**).

The noisy TFD, together with results produced by the non-adaptive LRE-based algorithm [20] and the proposed block-adaptive LRE algorithm is reported in Figure 3a,c,e.

The bird song signal has been collected in an environment of howling wind and rustling leaves (psithurism), by a bit depth of 16 bits per sample, with a sampling rate of 12 kHz. The noisy TFD, the outputs of the non-adaptive LRE-based algorithm [20] and the proposed block-adaptive LRE algorithm are reported in Figure 3b,d,f.

For both reported real-life signals, the non-adaptive LRE approach results in a significant loss of useful information compared to the proposed block-adaptive LRE algorithm.

In fact, for both considered signals, the non-adaptive LRE estimation applies an excessively strict criterion on signal components, as shown in Figure 3c,d.

The non-adaptive LRE criterion is determined by the worst noise conditions encountered over the entire measurement time. As a result, the non-adaptive LRE provides near-to-optimal denoising only for the most compromised parts of the signal TFD, with unnecessary useful information loss elsewhere. On the other hand, the block-partitioning treats sub-regions with less variable noise levels, which assures locally adequate *K*-means segmentation and efficient noise removal, with a contained loss of useful information (Figure 3e,f).

For real-life signals, an ideal, noise-free counterpart of the signal is not available, as well as a numerical assessment of the algorithms’ performance. However, the visual assessment suggests the method provides a more favorable trade-off between noise suppression and useful information loss when compared to the non-adaptive LRE approach.

This, however, needs to be corroborated by numerical evidence on synthetic data.

### 3.2. Simulation Results

The results provided by the proposed block-adaptive LRE algorithm are compared to those obtained by the non-adaptive LRE-based algorithm presented in [20] and the well-performing *K*-means ICI algorithm, which combines TFD *K*-means segmentation with an intersection of confidence intervals (ICI) statistical criterion on classes supports to recover useful information [8].

The performance quality is evaluated by means of the error-rate measure. The error rate is calculated by subtracting the denoised TFDs from the reference, noise-free TFD. The number of residual non-zero elements represents the error rate as a percentage of the N×M-dimensional set of observations.

The results are presented for two multi-component test signals for different SNRs.

The first of the test signals, which will be referred to as Sig 1, consists of two linearly frequency modulated components, with the addition of non-stationary white Gaussian noise.

The second signal, which will be referred to as Sig 2, presents two components, with sinusoidal and parabolic frequency modulations, respectively. Sig 2, which is embedded in additive non-stationary uniform white noise, and presents intersecting components. The signals’ parameters are reported in Table 1.

**Figure 4:**
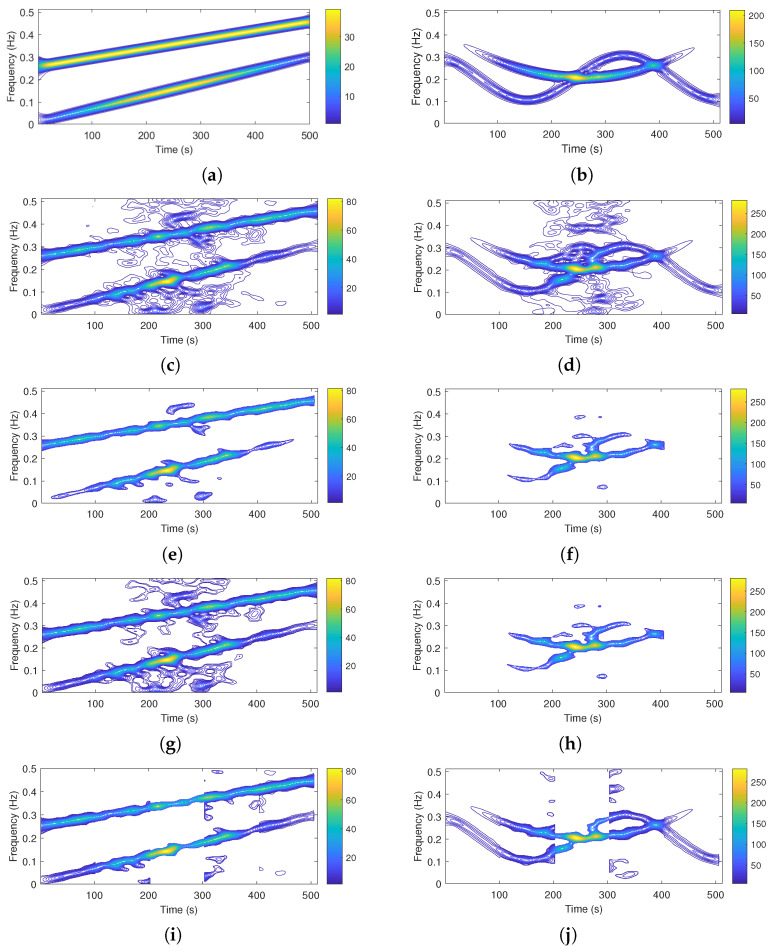
Noise-free TFD (**a**,**b**), noisy TFD (**c**,**d**), extracted signal components by the non-adaptive LRE algorithm (**e**,**f**), extracted useful information by the ICI method (**g**,**h**), and extracted useful information by the proposed block-adaptive LRE algorithm (**i**,**j**). Signals are referred to as Sig 1 (left column) and Sig 2 (right column).

The spectrograms of the noise-free and noisy signals (computed with a *Hamming* window with a duration of round (N/7) the information content extracted by the non-adaptive LRE algorithm, the *K*-means/ICI method, and the block-adaptive LRE algorithm are shown in Figure 4.

Table 2 reports the obtained results in terms of error rate (ER), and false negative (FN) estimates, representing the useful information damage. Results are based on 1000 independent noise realizations for the two test signals and four different SNRs. For the proposed algorithm, results are reported for three different values of Δt (building-block duration).

## 4. Discussion

As visible from Figure 4 and corroborated by numerical results in Table 2, the proposed adaptive method convincingly outperforms the non-adaptive LRE-based algorithm and the ICI method in terms of ER; even better results are obtained for both the test signals over the entire simulation set while maintaining stable performance with respect to the parameter Δt.

In terms of ER performance, improvement compared to the non-adaptive LRE-based algorithm spans from 10.76% (Δt = N/5, 6 dB) to 19.10% (Δt = N/9, 0 dB) in the case of *Sig 1*.

For *Sig 2*, the lowest error-rate improvement compared to the non-adaptive LRE-based algorithm is close to 25% (Δt = N/9, 3 dB), while the largest reaches up to 38% (Δt = N/5, 6 dB).

When compared to the ICI method, the proposed algorithm reduces the ER in the range of 4.19 (Δt = N/9, −3 dB) to 9.51% (Δt = N/9, 6 dB) in the case of *Sig 1*.

For *Sig 2* the ER reduction varies from 7.49% (Δt = N/9, 6 dB), to 24.94% (Δt = N/9, 0 dB).

Considering the FN component of the ER brings detailed insight into the performance of the compared methods.

Compared to the proposed method, the non-adaptive LRE-based algorithm presents significantly larger ER and FN estimates, suggesting that it provides a too strict criterion in the case of variable noise conditions, causing unjustified loss of useful information.

In terms of FN estimates, the ICI method performs similarly to the proposed algorithm, while maintaining a substantially larger ER. This is to indicate that the criterion applied by the ICI method is too concessive for signals embedded in non-stationary noise.

Partitioning of the TFD allows the proposed block-adaptive algorithm to enhance the sensitivity of the *K*-means amplitude segmentation since inside one time block near-to-stationary noise conditions are achieved. As a result, time blocks present lower noise levels and better-preserved components are spared from the strict criterion that is applied to blocks severely degraded by noise presence. This results in a generally better recovery of signal components while maintaining accurate denoising over different time blocks.

The partitioning does not add a significant computational burden. The computation of local entropy is in effect done here across the whole TFD, so the total computational cost is O(NMK), the same as the non-adaptive LRE approach. The block-based method presented here can be seen as iterative applications of non-adaptive LRE over smaller segments, so *K*-means is done multiple times but over smaller segments. The approximate *K*-means algorithm’s cost is controlled by an iteration count *I* and is linear in the size of the blocks. Thus, we apply *K*-means N/Δn times on M×Δn blocks with individual complexity O(MΔnIK), resulting in O(MNKI), asymptotically the same as for non-adaptive LRE.

The complexity of the 2-D entropy estimation [21], on the other hand, is $O(N^{5})$, which is impeditive for application on real-life signals and extensive simulations.

Concerning parameter tuning, the selection of *K*, Δt, and Δn should be discussed.

The number of classes *K*, analyzed in [24], is recommended to be in the range K=6…12. If chosen from the reported interval, the influence of the number of classes on the segmentation results is marginal.

As for the size of the building block length Δt, the obvious relation N>Δt>Δn is imposed, where Δn, is a constant in Equation (19) [20]. The size of the building block length Δt determines the degree of the algorithm’s adaptability. Values that are too large would result in the algorithm’s poor adjustment to the signal’s local conditions. For values that are too small, the windows would cause a loss of the TFD’s structural features, which are the core of the denoising process. However, both these extremes can be intended as loose constraints, since results are stable for independent ranges N/10<Δt<N/5 and N/50<Δn<N/25.

Thus, none of the parameters can be considered critical.

## 5. Conclusions

This paper presents a method for signal denoising in time-variable noise conditions. The denoising criterion is based on amplitude segmentation and LRE estimation over individual time building blocks of the signal spectrogram. By introducing the block-adaptive approach, the effects of noise non-stationarity are minimized.

In view of these considerations, and based on the reported results, the local estimation approach of the TFD’s structural features results in being beneficial for efficient denoising in the case of non-stationary noise.

Furthermore, in this work, we have considered only non-stationary signals with variable noise intensity over time. On the other hand, if signals were corrupted by colored noise with uneven frequency distribution, an extension of the methods’ adaptivity should be considered. Band-limited block LRE estimation, representing a multi-dimensional adaptive denoising method, will be the focus of our future work.

## Figures and Tables

**Table 1:** Parameters of the test signals from Figure 4.

	$\begin{array}{c} y\left(n\right)=\sum_{l=1}^{L}x_{l}\left(n\right)+w\left(n\right)\nu \left(n\right),\;x_{l}\left(n\right)=A_{l}\left(n\right)e^{j\Phi_{l}\left(n\right)}\\ L=2,w\left(n\right)=e^{-2\beta^{2}\frac{{\left(n-N/2\right)}^{2}}{{\left(N-1\right)}^{2}}},\;1\leq n\leq N,\;N=500 \end{array}$				
	β	$A_{1}\left(n\right)$	$A_{2}\left(n\right)$	$\Phi_{1}\left(n\right)$	$\Phi_{2}\left(n\right)$
*Sig 1*	1	1	$\begin{array}{c} e^{-\pi \frac{{\left(n-n_{0}\right)}^{2}}{T^{2}}}\\ n_{0}=250,\\ T=490 \end{array}$	$2\pi (195\times 10^{-6}n^{2}+249\times 10^{-3}n-76)$	$2\pi (283\times 10^{-6}n^{2}+9\times 10^{-3}n-21)$
*Sig 2*	3.5	1	$\begin{array}{c} 2e^{-\pi \frac{{\left(n-n_{0}\right)}^{2}}{T^{2}}},\\ n_{0}=250,\\ T=290 \end{array}$	$0.07N\sin(\frac{2\pi (n-35)}{0.7N}+1)+0.4\pi n-111$	$2\pi (10^{-6}n^{3}-8\times 10^{-4}n^{2}+0.4n)$

**Table 2:** Performance comparison for signals in Figure 4. Values are averaged from 1000 simulations of the signal with different noise realizations.

(a) Error Rate (%)						(b) False Negative (%)			
		ER, Sig 1				FN, Sig 1			
SNR		−3	0	3	6	−3	0	3	6
		Proposed method				Proposed method			
	N/5	13.55	11.86	10.28	9.86	12.24	11.11	9.25	9.54
Δt=	N/7	13.62	12.05	10.20	9.60	12.45	11.36	9.33	9.24
	N/9	13.94	11.81	10.42	9.51	12.94	11.13	9.61	9.42
		Non-adaptive LRE method				Non-adaptive LRE method			
		16.48	14.6	12.16	11.05	15.31	13.17	11.34	10.65
		ICI method				ICI			
		14.55	12.92	10.88	10.51	12.79	11.29	9.93	9.12
		ER, Sig 2				FN, Sig 2			
SNR		−3	0	3	6	−3	0	3	6
		Proposed method				Proposed method			
	N/5	11.08	9.98	9.08	8.21	9.89	8.75	8.10	7.35
Δt=	N/7	11.05	9.91	9.12	8.65	9.61	9.03	8.27	7.80
	N/9	11.14	9.72	9.38	9.01	10.00	8.76	8.31	7.90
		Non-adaptive LRE method				Non-adaptive LRE method			
		14.95	13.42	12.52	13.30	14.57	13.42	12.11	13.30
		ICI method				ICI			
		14.65	12.95	11.26	9.74	10.28	10.53	10.08	7.47

## Data Availability

The data presented in this study are available on request from the corresponding author.

## Abbreviations

The following abbreviations are used in this manuscript:

Abbreviations	
AWGN	Additive white Gaussian noise
TFD	Time-frequency distribution
DFT	Discrete Fourier transform
TF	Time-frequency
LRE	Local Rényi entropy
SNR	Signal-to-noise ratio
Nomenclature	
$A_{l}$	instantaneous amplitude of the *l*-th component
C	set of observations derived from a TFD
$C_{t}$	set of observations derived from one TFD building block
$C_{I}$	subset of TFD’s observations derived from useful information
$C_{k}$	*k*-th set of partitioned TFD’s observations
$C_{k,t}$	*k*-th set of partitioned observations from one TFD building block
$d_{k}$	closeness value between LRE estimates $H_{k}$ and $H_{k+1}$
$d_{k}$	closeness value between block-wise LRE estimates $H_{k,t}$ and $H_{k+1,t}$
*f*	continuous frequency
$f_{l}\left(t\right)$	instantaneous frequency of the *l*-th component
$f_{s}$	sampling frequency
*G*	time-lag kernel
$H_{k}$	Rényi entropy of the *k*-th TFD class
$H_{k,t}$	Rényi entropy of the *k*-th class, and *t*-th building block
*i*	smallest index of useful classes $\rho_{k}$
$i_{t}$	smallest index of building-block useful classes $\rho_{k,t}$
*L*	number of signal components
*K*	number of TFD classes
*l*	discrete lag
*m*	discrete frequency
*M*	number of frequency bins
*n*	discrete time
$n_{0}$	parameter defining the amplitude modulation time-shift
*N*	number of time samples
*p*	specific time instant
*t*	continuous time
*T*	parameter defining the signal component amplitude modulation variance
UI	extracted TFD useful information
$UI_{t}$	useful information extracted per one TFD building-block
*w*	non-constant positive noise amplitude modulation
$x_{l}$	*l*-th signal component
*x*	noise-free signal
*y*	noisy signal
α	Rényi entropy order
β	parameter defining the amplitude noise modulation variance
Δm	frequency resolution
Δn	duration of LRE estimation interval
Δt	duration of one TFD building block
ν	additive white noise
ρ	quadratic TFD
$\rho_{I}$	TFD of extracted useful information
$\rho_{k}$	*k*-th TFD class
$\rho_{k,t}$	*k*-th class of TFD building block
$\rho_{t}$	TFD building block
$\rho_{y}$	TFD of a noisy signal
τ	continuous lag
$\Phi_{l}$	instantaneous phase of the *l*-th component
